# Comparative serum proteomic analysis of a selected protein panel in individuals with schizophrenia and bipolar disorder and the impact of genetic risk burden on serum proteomic profiles

**DOI:** 10.1038/s41398-022-02228-x

**Published:** 2022-11-09

**Authors:** Mojtaba Oraki Kohshour, Nirmal R. Kannaiyan, August Jernbom Falk, Sergi Papiol, Urs Heilbronner, Monika Budde, Janos L. Kalman, Eva C. Schulte, Marcella Rietschel, Stephanie Witt, Andreas J. Forstner, Stefanie Heilmann-Heimbach, Markus M. Nöthen, Carsten Spitzer, Berend Malchow, Thorsten Müller, Jens Wiltfang, Peter Falkai, Andrea Schmitt, Moritz J. Rossner, Peter Nilsson, Thomas G. Schulze

**Affiliations:** 1 Institute of Psychiatric Phenomics and Genomics (IPPG), University Hospital, LMU Munich, Munich, Germany; 2 Department of Immunology, Faculty of Medicine, Ahvaz Jundishapur University of Medical Sciences, Ahvaz, Iran; 3 Department of Psychiatry and Psychotherapy, University Hospital, LMU Munich, Munich, Germany; 4 Department of Protein Science, SciLifeLab, KTH Royal Institute of Technology, Stockholm, Sweden; 5 International Max Planck Research School for Translational Psychiatry (IMPRS-TP), Max Planck Institute of Psychiatry, Munich, Germany; 6 Department of Genetic Epidemiology in Psychiatry, Central Institute of Mental Health, Medical Faculty Mannheim, Heidelberg University, Mannheim, Germany; 7 Institute of Human Genetics, University of Bonn, School of Medicine & University Hospital Bonn, Bonn, Germany; 8 Department of Psychosomatic Medicine and Psychotherapy, University Medical Center Rostock, Rostock, Germany; 9 Department of Psychiatry and Psychotherapy, University Medical Center Göttingen, Göttingen, Germany; 10 German Center for Neurodegenerative Diseases (DZNE), Göttingen, Germany; 11 iBiMED, Medical Sciences Department, University of Aveiro, Aveiro, Portugal; 12 Laboratory of Neuroscience (LIM27), Institute of Psychiatry, University of Sao Paulo, São Paulo, SP Brazil; 13 Department of Psychiatry and Behavioral Sciences, SUNY Upstate Medical University, Syracuse, NY USA; 14 Department of Psychiatry and Behavioral Sciences, Johns Hopkins University School of Medicine, Baltimore, MD USA

**Keywords:** Diagnostic markers, Human behaviour, Psychiatric disorders

## Abstract

The diagnostic criteria for schizophrenia (SCZ) and bipolar disorder (BD) are based on clinical assessments of symptoms. In this pilot study, we applied high-throughput antibody-based protein profiling to serum samples of healthy controls and individuals with SCZ and BD with the aim of identifying differentially expressed proteins in these disorders. Moreover, we explored the influence of polygenic burden for SCZ and BD on the serum levels of these proteins. Serum samples from 113 individuals with SCZ and 125 with BD from the PsyCourse Study and from 44 healthy controls were analyzed by using a set of 155 antibodies in an antibody-based assay targeting a selected panel of 95 proteins. For the cases, genotyping and imputation were conducted for DNA samples and SCZ and BD polygenic risk scores (PRS) were calculated. Univariate linear and logistic models were used for association analyses. The comparison between SCZ and BD revealed two serum proteins that were significantly elevated in BD after multiple testing adjustment: “complement C9” and “Interleukin 1 Receptor Accessory Protein”. Moreover, the first principal component of variance in the proteomics dataset differed significantly between SCZ and BD. After multiple testing correction, SCZ-PRS, BD-PRS, and SCZ-vs-BD–PRS were not significantly associated with the levels of the individual proteins or the values of the proteome principal components indicating no detectable genetic effects. Overall, our findings contribute to the evidence suggesting that the analysis of circulating proteins could lead to the identification of distinctive biomarkers for SCZ and BD. Our investigation warrants replication in large-scale studies to confirm these findings.

## Introduction

Schizophrenia (SCZ) and bipolar disorder (BD) are two severe, polygenic neuropsychiatric disorders caused by the complex interplay of multiple biological and environmental factors. The diagnostic criteria of SCZ and BD are still essentially based on the clinical evaluation of symptoms and signs; for instance, individuals with BD have better neuropsychological performance and fewer structural brain abnormalities than individuals with SCZ. However, the similarities in the clinical manifestations of SCZ and BD can lead to misdiagnosis, inappropriate therapeutic interventions, and poor outcomes [1–4].

Despite the lack of knowledge regarding the mechanisms that lead to these disorders, compelling evidence suggests that the immune system, particularly inflammation and autoimmunity, plays a role in the origin and disease course of mental disorders [5]. The most associated locus in SCZ genome-wide association studies (GWAS) maps to the major histocompatibility complex on chromosome 6, specifically the complement 4 A (C4A) locus [6, 7]. Moreover, increased circulating pro-inflammatory cytokine levels have been detected in individuals with BD, suggesting that immune system dysfunction may be involved in the pathophysiology of BD [8]. Likewise, studies in individuals with SCZ have reported a significant increase in the macrophage-derived circulating cytokines interleukin (IL)-1β, IL-6, and tumor necrosis factor α and the T helper 1-derived cytokines interferon γ and IL-12 in patients with acute relapse or first-episode psychosis [9].

In this context, identifying disease-associated, immunity-related proteins in the blood would represent a minimally invasive and cost-effective method that could contribute to (i) our understanding of the molecular mechanisms and pathways involved in SCZ and BD [10] and (ii) the discovery of disease-specific biomarkers that hold potential as predictors of disease risk, disease progression, and treatment response in SCZ and BD [8].

To achieve the full potential of blood biomarkers for better diagnosis and prognosis of these disorders, the discovery of such markers should be based on well-defined patient cohorts and cutting-edge high-throughput technologies [11–13]. Within this framework, the application of affinity properties simplifies the discovery and confirmation of the new biomarkers. Such an affinity-based approach is antibody-based microarray, a fast, and specific technology with high potential in proteomics that has attracted attention in biomarker research and patient stratification fields [14, 15].

Results from the largest GWAS on SCZ and BD also confirmed the high polygenicity of both disorders and their overlapping genetic liability [6, 16, 17]. The effect sizes of identified alleles derived from GWAS can be summed up to calculate a polygenic risk score (PRS), i.e., an individual estimate of genetic burden [18]. Although little is known about the association of the individual genetic load with specific proteomic signatures in peripheral blood in patients with SCZ and BD, a previous study suggested that SCZ-PRS and BD-PRS are associated with blood levels of CCL4 and ghrelin [19]. Therefore, the goals of the current study were i) to ascertain whether diagnosis-specific signatures exist in the circulating proteome that differentiate between individuals with SCZ and BD and healthy controls or between individuals with SCZ and those with BD and ii) to use PRS analyses to determine whether these proteomic profiles are influenced by the individual genetic burden for each disorder.

## Materials and methods

### Samples

This pilot study included 113 individuals with SCZ and 125 with BD, both diagnosed according to DSM-IV criteria, and 44 healthy controls. The patients were part of the multi-site German/Austrian longitudinal PsyCourse Study (www.psycourse.de) that collected deep phenotypic data and biomaterials from individuals with different psychiatric diagnoses. Controls were obtained from an ongoing study at the Department of Psychiatry, University Hospital Munich. Among them, individuals with neurological diseases affecting the central nervous system (e.g. psychiatric disorders, epilepsy, stroke, multiple sclerosis, dementia, meningitis and encephalitis, structural brain deficits, organic psychosis/mania) or other severe somatic comorbidities were excluded. All participants provided written informed consent. The study was performed in accordance with the principles of the Declaration of Helsinki and was approved by the ethics committee of the University Hospital Munich (Application number: 17–13).

The current analyses were based on the v4.1 version of the PsyCourse dataset [20]. The individuals with SCZ and BD were selected with the aim of matching the patient groups as closely possible regarding demographics and disease severity. Further details on the sample can be found elsewhere [21].

### Protein quantification by antibody suspension bead array

A panel of 95 serum proteins (Supplementary Table S1) was analyzed with a set of 155 antibodies in a high-throughput antibody-based assay in all the samples (cases and controls) following the same protocols. The protein panel was mainly selected with the aim to quantify the expression landscape of immune-related proteins in serum. Other common proteins in serum, such as apolipoproteins, and interesting candidates (e.g. Neuregulin 1 [NRG1], Erb-B2 Receptor Tyrosine Kinase 4 [ERBB4], and Vascular Cell Adhesion Molecule 1 [VCAM1]) derived from genetic studies in SCZ and BD were also included.

Multiplex protein profiling was performed by suspension bead array technology in combination with antibodies generated within the Human Protein Atlas (www.proteinatlas.org) [22], as previously described [23]. In short, crude serum was diluted 1:10, and the protein content was labeled with NHS-Biotin. In parallel, Human Protein Atlas antibodies against the selected proteins were covalently coupled to color-coded magnetic beads and afterwards combined to form a bead array. Labeled samples were diluted 1:50, heat treated for 30 min at 56 °C and then incubated with the bead array overnight. Beads were washed, and streptavidin-conjugated R-phycoerythrin was added for protein detection. The readout was performed with a Luminex FlexMap 3D and yielded a median fluorescent intensity (MFI) per bead and sample for reads above 50 beads. MFI was processed by antibody-specific probabilistic quotient normalization (AbsPQN) to minimize the influence of the background signal [24]. Log2 transformation, standardization, and removal of outliers above 3 SD were implemented in each protein quantification for downstream analyses.

### Calculation of PRS

Genotyping (Infinium PsychArray-24 BeadChip), quality control, and imputation (1000 Genomes Phase 3 reference panel) were performed as described elsewhere [25]. The latest GWAS in SCZ, BD, and SCZ-vs-BD were used as discovery datasets [6, 16, 17]. To obtain an individual estimate of SCZ, BD, and SCZ-vs-BD genetic risk burden, PRS were calculated with the “PRS continuous shrinkage” approach (PRS-CS; “auto” settings) to estimate the effect sizes of each genetic variant [26] and by summing up the weighted effect of each single nucleotide polymorphism (SNP) contributing to the PRS. PLINK 1.9 was used for PRS scoring [27].

### Statistical analysis

Logistic regression models were implemented in R version 3.6.3 (https://www.R-project.org/) to analyze the association of protein levels or the proteome-based PCs with diagnosis status, and linear models were used to ascertain the association of SCZ, BD, and SCZ-vs-BD polygenic load with the measured protein levels and the proteome-based PCs. Sex, age, duration of illness, ancestry principal components 1 and 2 (only in analyses involving PRS), diagnosis (in the linear model), and medication were used as covariates in all linear/logistic analyses. Medication was categorized as antipsychotics, antidepressants, mood stabilizers, and tranquilizers. The proportion of explained variance (both R^2^ and Nagelkerke’s pseudo-R^2^) was calculated by subtracting the effects of the covariates from the full model with PRS. Principal component analysis (PCA), an unsupervised feature transformation method, was used to reduce the dimensionality of our proteomic dataset and to investigate potential batch effects a priori using age- and sex- and medication- corrected residuals [28]. The performance of the models with diagnosis-associated predictors was assessed by 10-fold cross-validation by using the caret R package [29]. The comparison results were considered statistically significant if *p* < 0.05; false discovery rate (FDR) and Bonferroni corrections were used to adjust the results for multiple comparisons.

## Results

After quality control, 208 cases and 44 healthy controls remained. The demographic and psychopathological data of these 252 individuals are presented in Table 1. The two patient groups did not significantly differ in age, duration of disease, proportion of inpatients, or severity of depressive symptomatology. On the other hand, they did differ in the Positive and Negative Syndrome Scale (PANSS) general, positive, and negative scores and Young Mania Rating Scale (YMRS) score, and sex distribution. The healthy control group was significantly younger than both diagnostic groups and had a significantly lower proportion of women than the BD group. Compared to SCZ and control groups, the BD group had a notably larger percentage of women.

**Table 1:** Demographic and psychopathological data of study participants.

	SCZ	BD	HC	Test
Subjects (n)	108	100	44	—
Sex (%female)	40.7%	58%	34.1%	SCZ vs BD: χ-squared = 5.517; *p*-value = 0.0188 SCZ vs HC: χ-squared= 0.336; *p*-value=0.562 BD vs HC: χ-squared = 6.064; *p*-value = 0.014
Inpatient status (%inpatient)	36.1%	40%	—	χ-squared=2.564; p-value=0.463
Age (years, mean ± SD)	44.6 ± 13.9	46.2 ± 13.6	32.0 ± 9.9	SCZ vs BD: F-value=0.728; *p*-value=0.395 SCZ vs HC: F-value = 29.8; *p*-value = 1.94 × 10^−7^ BD vs HC: F-value = 39.06; *p*-value = 4.52 × 10^−9^
Duration of illness (years, mean ± SD)	14.9 ± 11.9	13.5 ± 12.2	—	SCZ vs BD: F-value=0.686; *p*-value=0.408
PANSS_Positive (mean ± SD)	12.8 ± 5.1	9.4 ± 2.9	—	SCZ vs BD: F-value = 30.58; *p*-value = 8.89 × 10^−08^
PANSS_Negative (mean ± SD)	13.9 ± 6.1	10.5 ± 3.9	—	SCZ vs BD: F-value = 20.51; p-value = 1.03 × 10^−05^
PANSS_General (mean ± SD)	26.6 ± 8.4	23.6 ± 6.5	—	SCZ vs BD: F-value = 7.857; *p*-value = 0.00557
YMRS (mean ± SD)	2.4 ± 4.3	4.2 ± 5.9	—	SCZ vs BD: F-value = 5.971; *p*-value = 0.0154
BDI-II (mean ± SD)	11.4 ± 10.7	12.6 ± 12.2	—	SCZ vs BD: F-value=0.469; p-value=0.494
IDS-C_**30**_ (mean ± SD)	14.4 ± 9.7	14.1 ± 11.2	—	SCZ vs BD: F-value=0.032; p-value=0.859

*BDI-II* Beck Depression Inventory, *IDS-C*_*30*_ Inventory of Depressive Symptomatology, *PANSS* Positive and Negative Syndrome Scale, *YMRS* Young Mania Rating Scale.

**Fig. 1:**
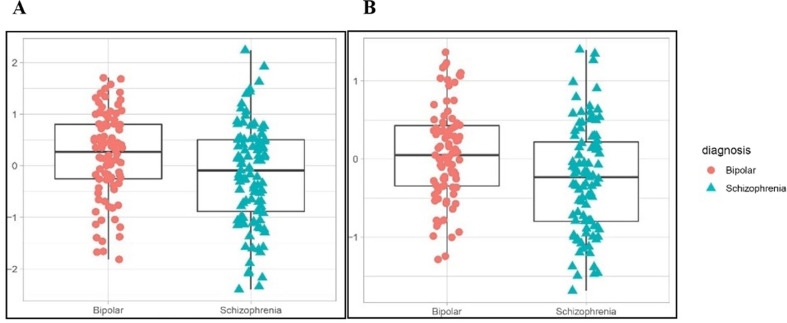
Box plots and data points for two significant serum proteins differentially expressed in patients with SCZ and BD. **A** Complement 9 **B** Interleukin 1 Receptor Accessory Protein. * y-axis: age-, sex-, duration of illness-, and medication-controlled residual values of each protein.

PCA revealed a remarkable batch effect between the patient and control groups (Supplementary Fig. S1*)* likely due to differences in the way serum samples were obtained and handled in the group of patients (PsyCourse Study) and controls (another independent study). Therefore, our group comparisons exclusively focused on the patient groups. After applying Bonferroni adjustment, we found a significant difference between individuals with SCZ and those with BD in two serum proteins (see Supplementary Table S2 for full results): complement C9 (C9; OR = 0.38; 95% CI, 0.23-0.63; Bonferroni-adjusted *p* value = 0.026; ∆Nagelkerke’s-*R*^2^ = 0.058) and Interleukin 1 Receptor Accessory Protein (IL1RAP; OR = 0.34; 95% CI, 0.19–0.60; Bonferroni-adjusted *p* value = 0.031; ∆Nagelkerke’s-*R*^2^ = 0.050). The level of both C9 and IL1RAP was higher in BD than in SCZ (Fig. 1).

**Fig. 2:**
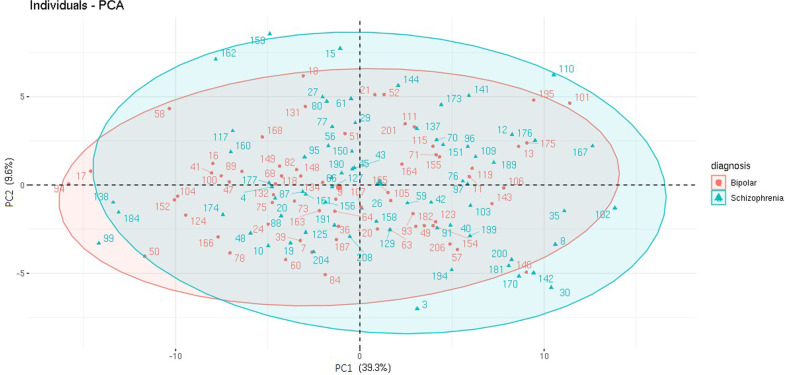
PCA scores plot. Levels of serum proteins in individuals visualized by diagnosis.

The final number of patients who had non-missing proteome information and thus were included in the PCA was 136. The demographic and clinical profile of this subsample was similar to that of the overall sample, except for the sex distribution in the BD group (Supplementary Table S3). PC1 explained 39.3% of the variance in the proteomic dataset in this study, and it was the only component that was significantly different between SCZ and BD (OR = 1.13; 95% CI, 1.04-1.26; Bonferroni-adjusted p value = 0.012; ∆Nagelkerke’s-*R*^2^ = 0.054; Fig. 2; Supplementary Fig. S2 and Table S4, S5).

Model performance parameters were calculated with a 10-fold cross-validation scheme. When C9, IL1RAP, or proteome-based PC1 were used as predictors, alone or combined, and diagnostic status was used as the predicted variable, none of the predictors yielded an area under the receiver operating characteristic curve (AUC-ROC) above 69% (Supplementary Fig. S3, Supplementary Table S6).

Regression analysis for effects of SCZ-PRS, BD-PRS, and SCZ-vs-BD–PRS on the levels of serum proteins revealed several nominal associations (*p* < 0.05, uncorrected) with each of the PRS. However, after Bonferroni and FDR corrections, no association remained significant. Likewise, none of these PRSs had a detectable influence on proteomic-derived PCs (Supplementary Tables S7-S12).

Center of recruitment and season of the year when the samples were collected did not show significant effects in the omnibus tests after multiple testing correction (Supplementary Tables S13, S14), so they were not included as covariates in linear/logistic models. Sex had a remarkable effect on leptin levels and age had an important influence on the levels of Insulin Like Growth Factor Binding Protein 2 and Complement C7 (Supplementary Tables S15, S16). Finally, univariate analyses of the effect of medication showed that, in general, large effects of pharmacological treatment are not to be expected, except for the effect of mood stabilizers on some few proteins (Supplementary Table S17).

## Discussion

The role of the immune system in mental disorders has been consistently highlighted in the literature, and inflammatory cytokines in particular have been linked in various ways to the risk of mental disease [30]. C9 is part of the complement system, the pore-forming component of the membrane attack complex, which forms pores in the membrane of targets [31]. IL1RAP is a necessary component of the IL1 receptor complex and initiates signaling processes that cause activation of IL1-responsive genes [32]. Interestingly, a methylation-wide association study comparing patients with SCZ and healthy controls reported a genome-wide CpG SNP in the IL1RAP locus in blood samples that replicated in a subsequent postmortem brain analysis, suggesting similar effects in both tissues [33].

In our sample, proteome-derived PC1 was also significantly associated with diagnosis. This finding suggests that the difference between SCZ and BD at the proteomic level involves a large number of proteins that are potentially implicated in particular pathways related to immune response.

Unfortunately, we were unable to analyze the levels of these proteins in the control samples because of batch effects, as described above. Therefore, the important question whether levels of these proteins are different in cases and controls requires follow-up investigations using a balanced sample ratio design.

Other studies with a similar design have been previously published. In 2015, Chan et al. introduced a panel of 26 serum biomarkers for identifying individuals at risk of developing SCZ [3], and in 2017, De Jesus et al. identified 13 unique, differentially abundant proteins (6 of them were included in our study) in the serum of individuals with BD that were not found in individuals with SCZ or other psychiatric illnesses or in controls [34]. A year later, Smirnova et al. reported 27 specific proteins for SCZ and 18 for BD (1 and 5 of them, respectively, were included in our study). These identified proteins play a major role in the immune response, among other processes [10]. A recent study from Mongan et al. convincingly showed that multivariable models, including clinical data and sets of circulating proteins, that are enriched in the complement system and the coagulation cascade are able to predict the transition to psychosis in at-risk individuals [35]. Importantly, for various reasons, such as technical differences, none of these studies observed C9 and IL1RAP as being differentially expressed in SCZ and BD, suggesting that our findings need to be validated in independent datasets of patients with a similar clinical and demographic profile as our patients. Two major changed pathways—complement and coagulation cascades—were found to be the most significant biological processes involved in both SCZ and BD [36, 37].

Despite being associated with diagnosis, C9 and IL1RAP levels and proteome-based PC1 had no predictive value regarding diagnostic status, as shown in the AUC-ROC analysis. These results indicate that studies with more sophisticated multivariable models and a design like the one used in the aforementioned study from Mongan et al [35]. are needed to improve performance in predicting disease status.

We did not observe significant effects of PRS on serum protein levels in individuals with SCZ and BD or on proteome-derived PCs, indicating that genetic burden has no effect on the proteins levels in our samples. However, our investigation was likely underpowered for detecting the usually modest effects of PRS in psychiatric diseases.

The concentration of serum proteins is tightly controlled in a normal state, but a wide range of diseases and treatments may result in changes in serum protein levels [38]. Previous evidence shows that the state of the disease and the medications affect protein serum levels. For example, BDNF levels are abnormally lowered in both manic and depressive phases of BD, and the reduced level in manic state rises following treatment;[39] or the inflammatory system is activated in SCZ and antipsychotics may have an impact on cytokines levels [40]. Our investigation is limited by the fact that not all patients had the same clinical status, and only the presence/absence of treatments at the time of assessment was taken into account as a covariate in the analyses. This limitation should be addressed in future studies with a similar design as ours. Another limitation of our study is the difference in the sex ratio between SCZ and BD groups.

Overall, the results of this pilot study confirm the relevance of the immune system in differentiating between SCZ and BD. However, our research raises doubts regarding the potential of proteomic biomarkers to predict both conditions, although more sophisticated models that incorporate both clinical and biological data may be useful. In this vein, the integration of multi-omics data holds promise for identifying reliable biomarkers in psychiatry. The results need independent replication in larger cohorts beyond a pilot study design. In this regard, our study and similar ones may lay the groundwork for large-scale studies and open new avenues for the discovery of effective theranostic approaches in neuropsychiatry that lead to a better predictive, preventive, and personalized medicine (PPPM) and eventually improve patients quality of life and reduce the socio-economic costs of mental illness [41, 42].

## Supplementary information

Supplemental material

Supplemental material
