# Supplementary material for: Comparative serum proteomic analysis of a selected protein panel in individuals with schizophrenia and bipolar disorder and the impact of genetic risk burden on serum proteomic profiles
Source: Transl Psychiatry. 2022 Nov 9;12:471. doi: 10.1038/s41398-022-02228-x (PMC9646817; doi:10.1038/s41398-022-02228-x)
Supplement: Supplementary file 1 — Supplemental material [file 41398_2022_2228_MOESM1_ESM.docx]

**Supplementary Material**

**Comparative serum proteomic analysis of a selected protein panel in individuals with schizophrenia and bipolar disorder and the impact of genetic risk burden on serum proteomic profiles**

**Table S1. List of 95 assayed serum proteins by antibody-based microarray in this study**

| **No.** | **Name** | **No.** | **Name** | **No.** | **Name** | **No.** | **Name** | **No.** | **Name** |
| --- | --- | --- | --- | --- | --- | --- | --- | --- | --- |
| **1** | **MIF** | **20** | **CCL5** | **39** | **CFP** | **58** | **HP** | **77** | **APOF** |
| **2** | **CD40LG** | **21** | **CEACAM5** | **40** | **IL15** | **59** | **CSF2** | **78** | **F7** |
| **3** | **C4BPA** | **22** | **FCN2** | **41** | **AXL** | **60** | **C4B** | **79** | **AGER** |
| **4** | **C8B** | **23** | **NRG1** | **42** | **APOD** | **61** | **IL1A** | **80** | **IL5** |
| **5** | **CFB** | **24** | **CCL11** | **43** | **IL12B** | **62** | **SERPING1** | **81** | **IL6** |
| **6** | **CFI** | **25** | **LTA** | **44** | **IL13** | **63** | **CFH** | **82** | **APOE** |
| **7** | **C1R** | **26** | **C1RL** | **45** | **IL7** | **64** | **APOB** | **83** | **CCL8** |
| **8** | **MBL2** | **27** | **ERBB4** | **46** | **IL4** | **65** | **TNF** | **84** | **C7** |
| **9** | **C1QA** | **28** | **VIP** | **47** | **CCL16** | **66** | **APOM** | **85** | **IL25** |
| **10** | **CSK** | **29** | **APOL1** | **48** | **C6** | **67** | **APOC1** | **86** | **ACE** |
| **11** | **IL6R** | **30** | **IL16** | **49** | **IL11** | **68** | **C4BPB** | **87** | **EGF** |
| **12** | **VWF** | **31** | **PTK2B** | **50** | **IGFBP2** | **69** | **IL17A** | **88** | **C9** |
| **13** | **VCAM1** | **32** | **C8A** | **51** | **BACE1** | **70** | **CFD** | **89** | **FCN3** |
| **14** | **APOH** | **33** | **MASP2** | **52** | **IL1B** | **71** | **IFNG** | **90** | **AVP** |
| **15** | **A2M** | **34** | **CD40** | **53** | **C8G** | **72** | **XCL2** | **91** | **APOA2** |
| **16** | **TNFRSF1B** | **35** | **LEP** | **54** | **C4A** | **73** | **CXCL8** | **92** | **APOC3** |
| **17** | **TNC** | **36** | **IL3** | **55** | **APOA1** | **74** | **KITLG** | **93** | **TNIK** |
| **18** | **ROCK2** | **37** | **IL1RAP** | **56** | **CCL18** | **75** | **APOC4** | **94** | **aAlbumin** |
| **19** | **PPBP** | **38** | **PPY** | **57** | **TGFB1** | **76** | **IL10** | **95** | **hIgG** |


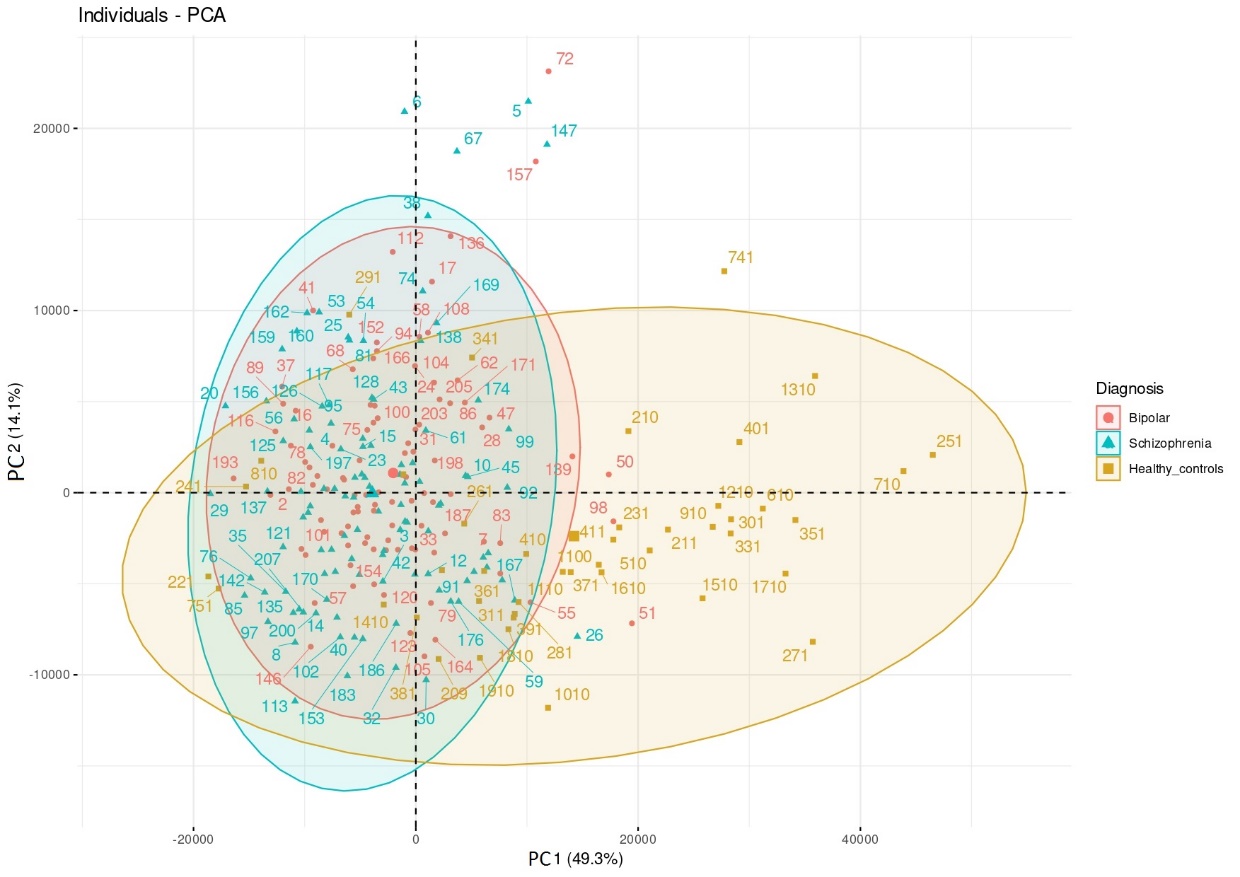
**Figure S1. PCA scores plot visualization of the batch effects between the patient and control groups**

**Figure S2. Scree plot of principal component analysis (PCA) of serum proteins levels in patients**

**
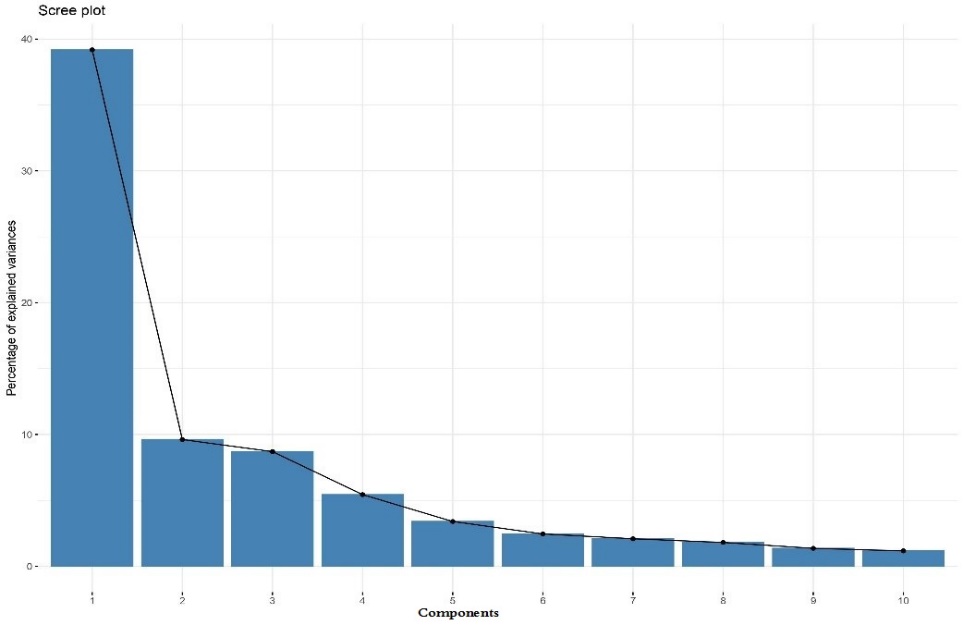
**

**Figure S3. AUC-ROC plot using C9 levels, IL1RAP levels, proteome-based PC1, and their combination as predictors and diagnostic status (SCZ/BD) as predicted variable. These analyses were carried out using a 10-fold cross-validation design.**

**
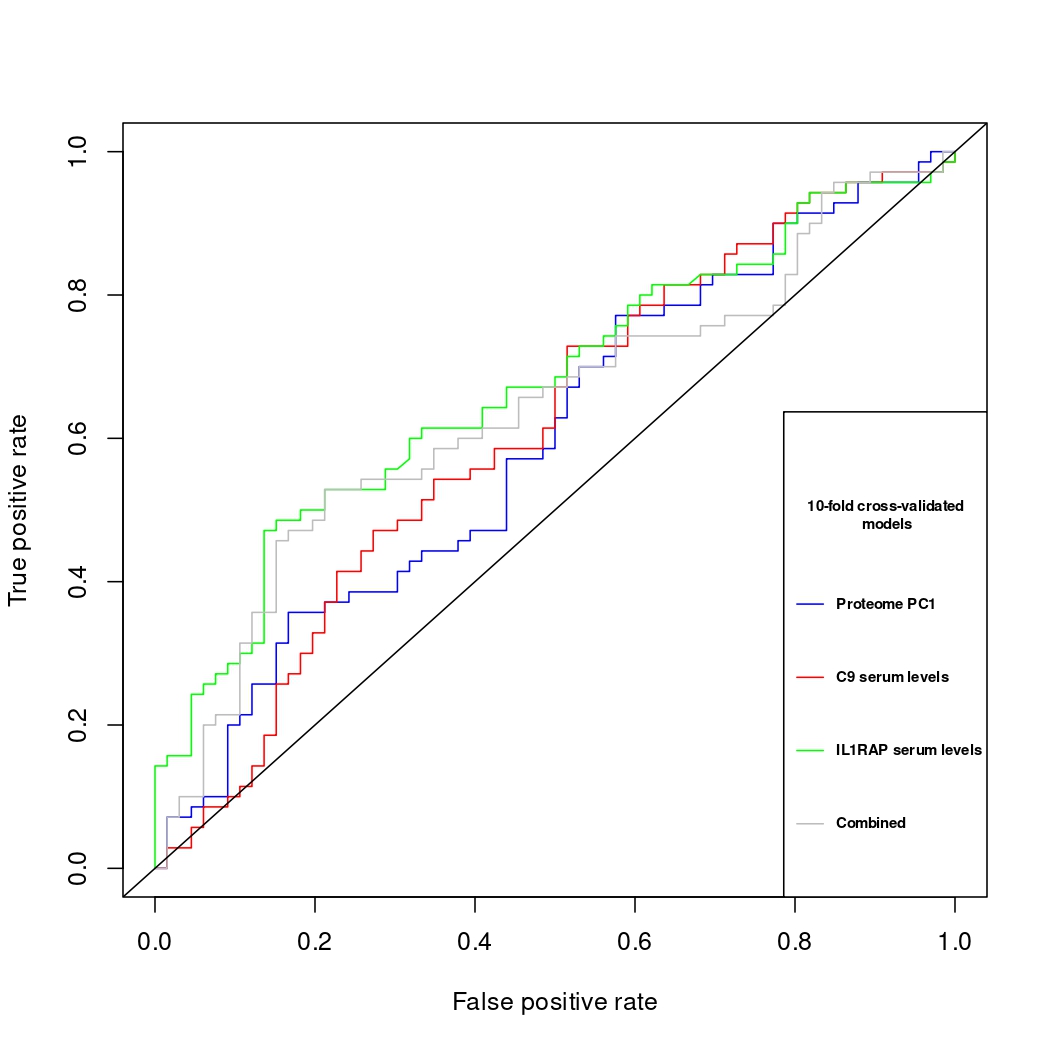
**
